# Continuous peri-operative glucose monitoring in noncardiac surgery: A systematic review

**DOI:** 10.1097/EJA.0000000000002095

**Published:** 2024-11-07

**Authors:** Alessandro Putzu, Elliot Grange, Raoul Schorer, Eduardo Schiffer, Karim Gariani

**Affiliations:** From the Division of Anaesthesiology, Department of Anaesthesiology, Pharmacology, Intensive Care and Emergency Medicine, Geneva University Hospitals (AP, EG, RS, ES), Faculty of Medicine, University of Geneva (ES) and Division of Endocrinology, Diabetes, Nutrition and Therapeutic Patient Education, Department of Medical Specialties, Geneva University Hospitals, Geneva, Switzerland (KG)

## Abstract

**BACKGROUND:**

Glucose management is an important component of peri-operative care. The usefulness of continuous glucose monitoring (CGM) in noncardiac surgery is uncertain.

**OBJECTIVE:**

To systematically assess the glycaemic profile and clinical outcome of patients equipped with a CGM device during the peri-operative period in noncardiac surgery.

**DESIGN:**

Systematic review.

**DATA SOURCES:**

Electronic databases were systematically searched up to July 2024.

**ELIGIBILITY CRITERIA:**

Any studies performed in the peri-operative setting using a CGM device were included. Closed-loop systems also administering insulin were excluded. Analyses were stratified according to diabetes mellitus status and covered intra-operative and postoperative data. Outcomes included glycaemic profile (normal range 3.9 to 10.0 mmol l^−1^), complications, adverse events, and device dysfunction.

**RESULTS:**

Twenty-six studies (1016 patients) were included. Twenty-four studies were not randomised, and six used a control arm for comparison. In bariatric surgery, diabetes mellitus patients had a mean ± SD glucose of 5.6 ± 0.5 mmol l^−1^, with 15.4 ± 8.6% time below range, 75.3 ± 5.5% in range and 9.6 ± 6.7% above range. During major surgery, diabetes mellitus patients showed a mean glucose of 9.6 ± 1.1 mmol l^−1^, with 9.5 ± 9.1% of time below range, 56.3 ± 13.5% in range and 30.6 ± 13.9% above range. In comparison, nondiabetes mellitus patients had a mean glucose of 6.4 ± 0.6 mmol l^−1^, with 6.7 ± 8.4% time below range, 84.6 ± 15.5% in range and 11.2 ± 4.9% above range. Peri-operative complications were reported in only one comparative study and were similar in CGM and control groups. Device-related adverse events were rare and underreported. In 9.21% of cases, the devices experienced dysfunctions such as accidental removal and issues with sensors or readers.

**CONCLUSION:**

Due to the limited number of controlled studies, the impact of CGM on postoperative glycaemic control and complications compared with point-of-care testing remains unknown. Variability in postoperative glycaemic profiles and a device dysfunction rate of 1 in 10 suggest CGM should be investigated in a targeted surgical group.

**KEY POINTS**

- This systematic review of the use of continuous glucose monitoring (CGM) during the peri-operative period included 26 studies and 1016 patients.
- CGM detected significant variability in glucose levels across various surgical specialties that depended on diabetic status.
- Device-related adverse events were rare, although device dysfunction occurred in approximately 10% of cases.
- Due to the limited number of controlled studies, the impact of CGM on postoperative glycaemic control and complications, compared with point-of-care testing, remains unclear.

## Introduction

Effective glucose management is an important element of peri-operative care. There is a large body of evidence indicating that peri-operative hyperglycaemia is associated with adverse outcomes such as prolonged hospital stay, postoperative infection, readmission or mortality in patients with and without diabetes.^1–3^

In the peri-operative period, hyperglycaemia in diabetic patients and stress hyperglycaemia in individuals not previously known for diabetes are commonly encountered. The magnitude of peri-operative hyperglycaemia will depend on several factors, such as the type of surgery, previous diabetes control, infection and other complications.^4^

Hypoglycaemia is associated with cognitive deficit, adverse cardiovascular events and potential death.^5^ Various risk factors have been associated with the development of peri-operative hypoglycaemia. These include fasting, low preoperative fasting glucose, diabetes for more than 10 years, low body mass index (BMI), use of sulfonylureas and other insulin secretagogues, previous episodes of hypoglycaemia, advanced age, preoperative administration of subcutaneous insulin and prolonged surgery.^6–8^

Peri-operative blood glucose monitoring, therefore, remains an important issue not only for diabetic patients but also for nondiabetic patient groups at increased risk of postoperative complications.

There are several tools available for peri-operative glycaemic monitoring, the most frequently used being point-of-care (POC) glucometers using capillary blood, which are widely available and low-cost. However, some peri-operative factors such as peripheral oedema or severe hypotension may affect device reliability and iterative testing can be time-consuming.^9–13^ Furthermore, repetitive blood sampling using a needle or fingerprick can be uncomfortable for some patients. Arterial blood gas analysis and traditional laboratory testing are alternatives to the monitoring of blood glucose.^10^

The advent of continuous glucose monitoring (CGM) has revolutionised the field of diabetology.^14^ Several studies have shown an advantage of CGMs compared with self-monitoring of blood glucose, mainly in type 1 diabetes but also for some patients with type 2 diabetes.^15,16^

There is growing interest in the benefits of CGMs outside of the usual ambulatory setting. The accuracy of CGM compared with POC testing has been satisfactory in inpatients and in the critically ill.^17,18^ Furthermore, various CGM implementation strategies have been suggested in the intensive care unit,^18^ but changes in illness acuity necessitate ongoing assessment of sensor function.^13,19^ Conversely, the potential role of CGM in noncardiac surgery remains unclear. Therefore, we aimed to systematically review the available evidence to describe the role of CGM on glycaemic control and its potential associations with significant clinical outcomes in the peri-operative period for noncardiac surgery patients.

## Materials and methods

This systematic review followed a pre-established protocol published in the PROSPERO database (CRD42023448990) using the Cochrane methodology.^20^ Reporting was aligned with the Preferred Reporting Items for Systematic Reviews and Meta-Analysis (PRISMA) guidelines (PRISMA 2020 checklist, Supplementary Table S1).^21^

### Systematic search

Two investigators (AP and EG) independently searched MEDLINE, EMBASE and the Cochrane Central Register of Clinical Trials for appropriate articles from inception to 6 July 2024. The search strategies are reported in the Supplementary Methods S1. For unpublished trials, we searched the National Institute of Health Clinical Trials Register (ClinicalTrials.gov). Bibliographies of retrieved studies and of relevant reviews were also screened for additional publications. No language restriction was enforced, and studies published only as abstracts were also included.

### Study selection

Studies were identified through examination of abstracts by two authors (AP and EG) and collected as full-text articles if potentially relevant. Eligible studies met the following PICOS criteria: patient group: adults undergoing noncardiac surgery; intervention: use of any CGM device; comparison intervention: any control intervention or lack of control group; outcome: see below; study design: observational and interventional studies.

We excluded case reports, studies with less than 10 patients, studies with overlapping patient groups already included in a previously selected article, and studies employing a closed-loop system (artificial pancreas) considered as co-intervention. Two authors (AP and EG) independently assessed selected studies for the final analysis. Any disagreements were resolved by consensus between the two authors.

### Data abstraction

Data extraction and entry was performed by one author and a second author verified the data, with divergences resolved by consensus (AP and EG). Sources of significant clinical heterogeneity were identified and recorded. These included study design, patient characteristics, CGM device type, intervention and control regimen. Corresponding authors were not contacted in case of missing outcome data.

We transformed results reported as interquartile or range into standard deviation using the formula recommended by the *Cochrane Handbook*.^20^ Medians were converted into means using a formula suggested for skewed outcomes.^20,22^ We excluded results with insufficient data to allow calculation of mean or standard deviation.

### Outcomes

The CGM glycaemic profile was characterised using some key metrics: time below range (TBR), time in range (TIR), time above range (TAR) and the coefficient of variation. TBR reflects the duration spent with blood glucose levels below the target range, indicating an increased risk of hypoglycaemia. TIR represents the percentage of time spent within the optimal glycaemic range. TAR highlights periods when blood glucose levels exceed the recommended range. Coefficient of variation is calculated by dividing the glucose standard deviation by the mean glucose.

The primary outcomes were mean glucose level, TBR, TAR, TIR and coefficient of variation. The outcome definitions were as reported from each study and were listed in the supplementary material (Supplementary Table S2). The secondary outcomes included various clinical endpoints comprising postoperative mortality, myocardial infarction, acute kidney injury and stroke. Post hoc tertiary outcomes were device-related adverse events (DRAE) and device dysfunction. Adverse events reported to be related to the CGM device were included in DRAE. Device dysfunction was defined as any event necessitating the replacement of the device (excluding replacement for MRI) or any event causing relevant missing data. Outcomes reporting by each study are listed in Supplementary Table S3. Study-reported outcomes are listed in Supplementary Table S3.

### Methodological quality

Two authors (AP and EG) independently assessed the risk of bias of each study and consensus was reached in case of divergences. For the nonrandomised studies, the methodological index for nonrandomised studies (MINORS) tool was used (Supplementary Methods S2).^23^ The overall judgement was categorised as good, fair or poor.

For randomised controlled trials (RCTs), the risk of bias was assessed by using the Cochrane Risk of Bias 2 tool. The assessment was performed at the primary outcome level. The overall risk-of-bias judgement was categorised as low risk of bias, some concerns and high risk of bias.

### Conflict of interest

Two authors (EG and AP) assessed potential financial and nonfinancial conflicts of interest. The methodology is reported in the supplement (Supplementary Methods S3). Each study was categorised according to the following categories: ‘notable concern about conflict of interest’, ‘no notable concern about conflict of interest’ or ‘unclear concern about conflict of interests’.

### Statistical analysis

The data from intra-operative and postoperative periods were stratified according to diabetes status: diabetes mellitus, nondiabetic/prediabetes (non-DM), and mixed. The analysis was further detailed regarding major surgical specialties: bariatric surgery, nonbariatric major surgery, pancreatic surgery (excluding pancreas transplantation) and transplantation surgery. Preoperative data were excluded from the analysis whenever possible. The planned meta-analysis and head-to-head comparisons were not performed because of the scarcity of comparative studies. The weighted percentage means of the primary outcomes were computed. The weights were the sample size of each study normalised to sum to one. The standard deviation of each study mean was computed and considered as an index of dispersion between studies. The analysis was performed using R version 4.3.2 using package 'stats’ and ‘dplyr’ (R Core Team (2023). _R: A Language and Environment for Statistical Computing_. R Foundation for Statistical Computing, Vienna, Austria (https://www.R-project.org/). A qualitative description of secondary and tertiary outcomes was performed due to underreporting and heterogeneity. Protocol deviations are reported in Supplementary Table S4.

## Results

### Systematic search

**Fig. 1:**
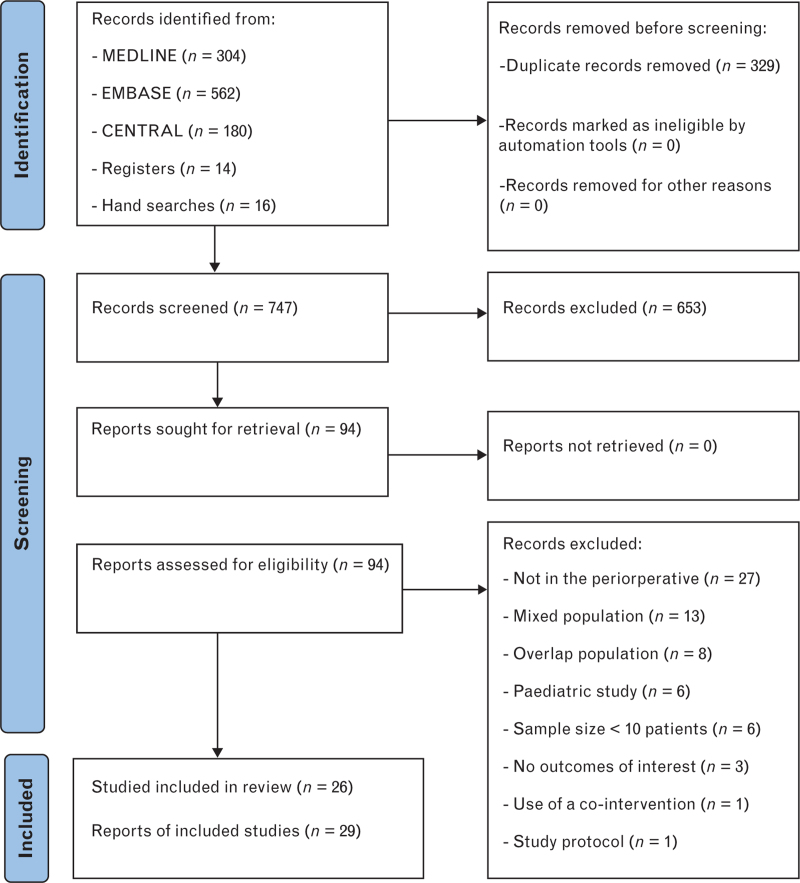
Flow diagram for the selection of studies.

The systematic search produced 1076 potential titles and abstracts from online databases and hand search (Fig. 1). Ninety-four articles were identified for review, and after exclusion of inadequate reports (Supplementary Table S4), we included 29 reports of 26 studies with a total of 1016 patients.^24–52^

### Study characteristics

The characteristics of the included studies are shown in Table 1 and Supplementary Table S6. Twenty-four were nonrandomised trials of which four were comparative studies. Nineteen studies were prospective. Two studies were RCTs. All studies but one were single centre. Thirteen studies were performed in Europe, 10 in Asia, 2 in Oceania and 1 in the United States.

**Table 1:** Study characteristics

Study	Country	Sample size	Surgery type	Diabetic status	Diabetes prevalence	CGM device	CGM monitoring
Carlsson 2023	Denmark	70	Major surgery	Mixed	71%	Dexcom G6	Intra- and postoperative
Fagher 2023	Sweden	100	Pancreatoduodenectomy	Mixed	23%	FreeStyle Libre 1	Postoperative
Farmanov 2024	Russia	26	Total duodenopancreatectomy	Mixed	46%	Medtronic device	Postoperative
Hagerf 2023	Czech Republic	14	Major abdominal surgery needing ICU	Mixed	50%	Dexcom G6	Postoperative
Hagerf 2024	Czech Republic	65	Major abdominal surgery or transplantation needing ICU stay	Mixed	51%	Dexcom G6	Postoperative
Herzig 2022	Switzerland	44	Elective surgery	DM2	100%	Dexcom G6	Intra- and postoperative
Jabor 2023	Czech Republic	61	Major abdominal surgery needing ICU	NR	NR	Dexcom G6	Postoperative
Jo 2022	South Korea	25	Kidney transplantation surgery	Mixed	24%	NR	Pre-, intra-, and postoperative
Kim 2022	South Korea	20	Metabolic surgery (LSG/DJB 50%, LSG 30%, LRYGB 15%, LBPD 5%)	DM2	100%	FreeStyle Libre	Pre- and postoperative
Leung 2023	China	83	Total knee arthroplasty	Mixed	27%	FreeStyle Libre	Postoperative
Maeda 2019	Japan	20	Total knee or hip arthroplasty	DM2 or pre-diabetes	60%	iPro 2	Postoperative
Mao 2021	China	18	Pancreatic or palliative abdominal surgery	Non-diabetes	0%	FreeStyle Libre	Postoperative
Mittal 2015	UK	26	Pancreas transplantation surgery with or without kidney transplantation	Mixed	NR	iPro 2	Postoperative
Munekage 2016	Japan	15	Pancreatoduodenectomy or hepatectomy needing ICU	NR	NR	iPro 2	Postoperative
Poljakova 2013	Czech Republic	17	Lower extremity vascular or orthopaedic surgery	DM2 or pre-diabetes	94%	Guardian REAL-Time	Pre-, intra-, and postoperative
Price 2023	USA	76	Surgery under general anaesthesia of at least 3 h expected duration	DM1 or DM2	100%	Dexcom G6 or FreeStyle Libre 2.0	Intra- and postoperative
Shaban 2023	Australia	21	Kidney transplantation surgery	Mixed	19%	FreeStyle Libre	Postoperative
Tripyla 2020	Switzerland	20	Major abdominal surgery	Diabetes or pre-diabetes	75%	Dexcom G6	Pre-, intra-, and postoperative
Turquetil 2021	France	31	Metabolic surgery (LRYGB 77%, LSG 23%)	DM2	100%	FreeStyle Libre	Pre-, intra-, and postoperative
Wang 2021	China	49	Metabolic surgery (LSG 100%)	Mixed	31%	MiniMed	Pre- and postoperative
Wysocki 2019	Poland	32	Metabolic surgery (LSG 56%, LRYGB 44%)	Mixed	50%	FreeStyle Libre	Pre-, intra-, and postoperative
Wysocki 2024	Poland	33	Metabolic surgery (LSG 100%)	DM2	100%	FreeStyle Libre	Pre-, intra-, and postoperative
Yin 2022	China	55	Amputation surgery	Mixed	87%	Medtronic device	Pre-, intra-, and postoperative
Yip 2014	New Zealand	21	Metabolic surgery (LRYGB 55%, LSG 45%)	DM2	100%	MiniMed MMT-7102	Pre- and postoperative
Yong Jin 2019	South Korea	31	Kidney or liver transplantation surgery	Mixed	19%	iPro 2	Postoperative
Zhang 2024	China	31	Esophagectomy	Non-diabetes	0%	MicroTech AiDEX model G7	Pre-, intra-, and postoperative

CGM, continuous glucose monitoring; DM, diabetes mellitus; ICU, intensive care unit; LSG/DJB, laparoscopic sleeve gastrectomy with duodenojejunal bypass; LSG, laparoscopic sleeve gastrectomy; LRYGB, laparoscopic Roux-en-Y gastric bypass; LBPD, laparoscopic biliopancreatic diversion; NR, not reported.

The mean age ranged between 35 and 70 years old and the proportion of women ranged between 14 and 82%. The types of procedures were varied, including seven studies of patients benefiting from major abdominal surgery, six from bariatric surgery (66% sleeve gastrectomy, 28% laparoscopic Roux-en-Y gastric bypass), three from pancreatic surgery, four from orthopaedic surgery, three from transplantation surgery (one pancreas transplantation),^38^ and three from miscellaneous surgery.

Diabetes prevalence ranged between 0 and 100% and mean BMI ranged between 21.2 and 47.5 kg m^−2^. Eight studies reported the use of a standardised peri-operative insulin protocol.

In terms of CGM device types, Freestyle Libre and Dexcom G6 were used in nine and seven studies respectively, iPro2 in four studies, and MiniMed in two studies. The Guardian REAL-Time, Guardian Connect, MicroTech AiDEX G7 and a nonspecified Medtronic device, were each used in one study. The estimated duration of CGM monitoring ranged between 1 and 28 days.

The most common position of the device was the dorsal side of the upper arm (414 patients), followed by the infraclavicular region (140 patients) and the abdomen (87 patients). The position of the devices was not clearly reported in 10 studies.

### Methodological quality

Twenty-four studies were nonrandomised and were evaluated with MINORS. Overall, 5 studies were evaluated to have fair methodological quality, whereas 19 had poor methodological quality (Supplementary Table S6).

Two studies were randomised: one RCT was judged to be at low risk of bias,^36^ and its sub-analysis was considered at high risk of bias due to the methodology not being defined a priori.^43^ The second RCT was judged to be at high risk of bias (Supplementary Figure S1).^51^

### Conflict of interest

Information on the funding sources and author financial conflicts of interest are reported in Supplementary Table S7. Overall, 16 studies were reported as having no notable concern about conflict of interest, 7 studies had a notable concern,^32,33,36,40,43,45,47,51^ and 3 studies were reported as having an unclear concern.^28,29,34^

Three studies reported financial support from a company producing CGM devices.^36,40,45^ In two cases, it was clearly reported that the company had no role in the study's design, conduct, analysis, or report.^36,45^

Seven studies had at least one author reporting a potential financial conflict of interest with companies related to the CGM industry.^32,33,36,40,45,47,51^ In total, 15 out of 77 authors (19%) of these studies had a conflict of interest. In two cases, it was the corresponding author.^33,47^

Seven studies were found to have nonfinancial conflicts of interest, with the reasons being multiple publications or acknowledged expertise within CGM, diabetes, or peri-operative monitoring.^24,28,33,36,38,47,52^

### Glycaemic profile

The results are detailed in Table 2. Five studies reported that CGM readings were not blinded during the study, potentially influencing peri-operative care. These studies are excluded from the aggregate analysis.^31,37,39,44,49^

**Table 2:** Primary outcomes in different surgical specialties

Surgery	Characteristics	Studies (patients)	Glucose level (mmol l^−1^) mean ± SD	Studies (patients)	TBR (%)	Studies (patients)	TIR (%)	Studies (patients)	TAR (%)	Studies (patients)	CV (%)
Bariatric surgery	DM	6 (136)	5.6 ± 0.5	5 (109)	15.4 ± 8.6	6 (130)	75.3 ± 5.5	5 (109)	9.6 ± 6.7	1 (20)	20.1
	Non/pre-DM	1 (16)	4.6 ± 0.1	1 (16)	26.7 ± 1.0	1 (16)	63.4 ± 0.2	1 (16)	7.6 ± 1.0	NR	–
	Mixed	6 (183)	5.3 ± 0.6	5 (156)	18.1 ± 8.4	6 (177)	72.6 ± 6.8	5 (156)	8.8 ± 5.8	1 (20)	20.1
Major surgery	DM	2 (71)	9.6 ± 1.1	2 (71)	9.5 ± 9.1	3 (75)	56.3 ± 13.5	2 (71)	30.6 ± 13.9	1 (21)	26.2 ± 6.1
	Non/pre-DM	3 (69)	6.4 ± 0.6	3 (69)	6.7 ± 8.4	4 (91)	84.6 ± 15.5	2 (49)	11.2 ± 4.9	2 (49)	23.9 ± 9.9
	Mixed	11 (373)	8.3 ± 1.3	6 (179)	7.5 ± 8.1	8 (220)	73.4 ± 18.2	7 (267)	21.2 ± 11.6	5 (203)	29.1 ± 7.0
Pancreatic surgery	DM	1 (21)	10.7 ± 0.6	1 (21)	0.3 ± 0.2	1 (21)	46.6 ± 8.8	1 (21)	45.6 ± 8.5	1 (21)	26.2 ± 6.2
	Non/pre-DM	1 (15)	6.5 ± 0.7	1 (15)	10.9 ± 4.1	1 (15)	80.4 ± 3.0	1 (15)	8.6 ± 6.4	1 (15)	36.8 ± 3.9
	Mixed	4 (54)	8.8 ± 1.8	2 (36)	4.7 ± 5.9	3 (48)	66.8 ± 19.1	3 (42)	29.2 ± 18.6	3 (42)	30.4 ± 6.9
Transplant surgery	DM	NR	–	NR	–	1 (4)	32.5	NR	–	NR	–
	Non/pre-DM	NR	–	NR	–	1 (22)	83.2 ± 29.9	NR	–	NR	–
	Mixed	3 (97)	8.5 ± 1.0	1 (25)	8.0	1 (26)	75.4 ± 33.1	2 (66)	21.7 ± 7.5	2 (66)	35.5 ± 1.0

Data reported as mean ± standard deviation. CV, coefficient of variation; DM, diabetes mellitus; NR, not reported; TBR, time below range; TIR, time in range; TAR, time above range.

The mean glucose levels were higher in patients undergoing major surgery (diabetes mellitus 9.6 ± 1.1; nondiabetes mellitus 6.4 ± 0.6 mmol l^−1^) vs. bariatric surgery (diabetes mellitus 5.6 ± 0; nondiabetes mellitus 4.6 ± 0.1 mmol l^−1^).

TBR, defined in most studies as less than 3.9 mmol l^−1^ (70 mg dl^−1^), was higher in bariatric surgery (diabetes mellitus 15.4 ± 8.6%; nondiabetes mellitus 26.7 ± 1.0%) vs. major surgery (diabetes mellitus 9.5 ± 9.1; nondiabetes mellitus 6.7 ± 8.4).

The TIR was defined in most studies as glycaemic values between 3.9 mmol l^−1^ (70 mg dl^−1^) and 10.0 mmol l^−1^ (180 mg dl^−1^). It was higher for diabetic patients undergoing bariatric surgery (75.4 ± 5.5%) vs. major surgery (56.3 ± 13.5%). In nondiabetic patients, TIR was over 80% for major surgery as well as transplantation and pancreatic procedures vs. 63.4 ± 0.2% for bariatric surgery.

TAR, defined in most studies as values greater than 10 mmol l^−1^ (180 mg dl^−1^), was higher in diabetic patients undergoing major surgery (30.6 ± 13.9%) and pancreatic surgery (45.6 ± 8.5%). In the nondiabetic or prediabetic group, TAR was increased in bariatric surgery (7.6 ± 0.9%), pancreatic surgery (8.6 ± 6.2%), major surgery (12.4 ± 3.6%), and particularly transplantation surgery (21.7 ± 7.5%). The coefficient of variation was rarely reported and varied from 20.1 to 35.5 ± 1.0.

### Postoperative complications

No comparative study reported clinical outcomes predefined by our meta-analysis. One RCT of the use of CGM vs. closed-loop insulin delivery in mixed elective surgery reported similar rates of Clavien–Dindo complications in both groups: Clavien–Dindo II or higher: 12 of 22 vs. 14 of 22, Clavien–Dindo III or higher: 4 of 22 vs. 6 of 22.^36^

### Device-related adverse events

Eight studies reported DRAEs, but no study reported a definition for DRAE (Supplementary Table S8). Five studies reported not encountering any DRAE (0 of 227 patients).^36,37,45–47^ One study reported self-limited bleeding, mild pruritus and skin irritation resolving spontaneously in 2 of 20 patients.^40^ Another study found that 2 of 65 patients had subcutaneous haematomas at the sensor insertion site, with degraded sensor function.^51^ A third study reported that 2 of 42 patients were unwilling to reimplant the sensor after pressure damage.^50^ We found no reports of serious or life-threating DRAEs.

### Device dysfunction

Thirteen studies reported CGM dysfunction data, and no study defined CGM dysfunction (Supplementary Table S8). The devices were subject to accidental removal and sensor or reader dysfunction. Two studies (37 patients) reported the absence of any device dysfunction, whereas 11 studies reported the presence of dysfunctions. Overall, about 56 of 608 patients (9.21%) experienced device dysfunction.

Troubleshooting involved either sensor replacement or exclusion of the data. In the studies where replacement was used and reported, 13 sensors were replaced in 165 patients. Thirty-four patients were withdrawn from the studies because of dysfunction issues.

The possibility of electromagnetic interference from surgical cauterisation was mentioned in five studies. Two of those studies placed the devices preoperatively, and some of these devices failed to function after warm-up, potentially because of electric interference.^40,45^ Two studies placed the devices before surgery and did not report any electromagnetic interference (0 of 40 devices).^37,46^ One study placed the devices in the postanaesthesia care unit to avoid this problem.^42^

## Discussion

This systematic review examines the role of CGM in noncardiac surgery. Due to the limited number of controlled studies, we were unable to evaluate the impact on postoperative glycaemic control and complications when comparing CGM with alternative strategies like POC testing. Notably, glycaemic profiles varied significantly across different procedures and in patients with or without diabetes. The results highlight that while adverse events are uncommon and typically mild, the chance of device dysfunction is up to 1 in 10.

The duration of CGM monitoring varied from 1 to 28 days, demonstrating its applicability for both short and extended postoperative periods. The types of CGM devices used, mainly Freestyle Libre and Dexcom G6, were implanted on the dorsal side of the upper arm, as well as the infraclavicular region and the abdomen.

### Significance of the findings

Adverse events and device dysfunctions were rarely reported and not uniformly defined across studies. About 10% of the devices experienced some dysfunction, including accidental removal and sensor or reader issues. The potential interference of surgical electrocautery with CGM devices highlights a specific concern for their reliability in the operating room,^53^ but this issue remains mostly undocumented in actual noncardiac surgery studies.

The glycaemic profile of noncardiac surgery patients varied across different types of procedures. Bariatric surgery is well known for its glucose-lowering effects and is the surgical setting for most published studies of peri-operative CGM.^54^ Approximately one-third of these patients have diabetes, and as many as half can achieve remission from type 2 diabetes a few years after the surgery, with CGM recordings suggested as one of the predictors.^33^ In the first few days after surgery, the specific re-alimentation protocol and changes in gastrointestinal physiology can be challenging, and this is reflected by a unique CGM pattern. The mean glucose level was below 6 mmol l^−1^, with TBR about 15% in diabetic patients and 25% in nondiabetic patients, indicating hypoglycaemia to be common in these patients.

Major nonbariatric surgery is a setting where significant surgical stress is expected, leading to dysglycaemia. Our analysis found that in nondiabetic patients, mean glucose values averaged 6.3 mmol l^−1^ with about 80% of samples within the normal glycaemic range. In patients with diabetes mellitus, mean glucose values were 9 mmol l^−1^, with approximately 60% of samples from people with diabetes mellitus within the normal glycaemic range.^47^ This suggests that significant durations of both hypoglycaemia and hyperglycaemia are detected in a high proportion of patients. Some major procedures like pancreatectomy or transplants are at higher risk of dysglycaemia, where an insulin-depleted status is induced, or higher doses of steroids are administered.

This variability emphasises the necessity of individualised glycaemic control and the potential of CGM in peri-operative glucose management in a targeted surgical group.

### Peri-operative glucose monitoring and continuous glucose monitoring

Monitoring and treatment of blood sugar levels in the peri-operative period is primarily aimed at diabetic patients, particularly in those patients receiving subcutaneous or intravenous insulin. Beyond the specific peri-operative use of CGMs, it remains to be determined which nondiabetic patients should require close monitoring of blood glucose levels.^55^ However, the evaluation of hyperglycaemia and hypoglycaemia in different patient groups indicates that both conditions are significantly associated with worse clinical outcome.^1–3,56^

Close blood glucose monitoring can be a substantial and time-consuming work.

Measuring blood sugar conventionally with a POC glucometer can take up to 5 min per sample.^57^ In this context, the use of CGM could prove useful to reduce the workload of operating room personnel. CGM may display other advantages, as it can identify hyperglycaemia or hypoglycaemia episodes earlier and allow rapid intervention to avoid dysglycaemic episodes, reducing peri-operative glucose variability.^4^ It has also been suggested that CGM may predict mortality: in critically ill patients monitored with CGM, the risk of in-hospital mortality increased incrementally with every 10% increase in TAR with a glucose threshold of 10.5 mmol l^−1^.^58^

The use of CGMs presents certain limitations, particularly during the peri-operative period. First of all, the precision of CGM is very significantly reduced when glycaemic values fall outside the range of 2.2 to 22.2 mmol l^−1^ (40 to 400 mg dl^−1^) for the most recent devices.^59^ However, an excursion of blood glucose values outside of this interval remains a rare event in the surgical context, especially elective surgery and therapeutic interventions should be initiated before reaching those values. In addition, some drugs such as paracetamol and ascorbic acid could interfere with sensor readings and may produce falsely elevated or decreased glucose values.^13^

In critically ill adults, CGM has been reported to be accurate and safe in multiple studies, even in those with shock requiring norepinephrine.^13,60,61^ However, ongoing assessment of sensor function is crucial in settings where acute clinical changes may affect device reliability. These changes include oedema, device compression and severe hypoperfusion.^13^ Furthermore, some studies have reported a CGM device failure rate of around 12% and accidental removal rates of up to 33% in a critical care setting.^60,61^

Even though a large comprehensive validation study is missing in noncardiac surgery, some studies have suggested good accuracy of CGM glucose readings in comparison to POC in various noncardiac surgery settings.^25,32,37,40,50,51^

Conflict of interest and the cost of CGM devices are also critical considerations. Two Cochrane systematic reviews found that conflicts of interest or industry-sponsored studies had more favourable conclusions.^62,63^ Financial support from CGM companies in some studies raises questions about potential biases, while the high cost of these devices could reduce their accessibility and widespread adoption in clinical practice.

Finally, the distinction between intensive glucose monitoring and intensive glucose management is crucial. Although CGM provides detailed glucose profiles, it does not inherently equate stringent glucose control, emphasising the need for comprehensive management strategies that extend beyond mere monitoring. This is the main reason we excluded studies that did not blind the CGM readings from our aggregate analysis. Currently, there is still a lack of global consensus on peri-operative glycaemic targets. Some guidelines recommend a level less than 10.0 mmol l^−1^, some less than 12.0 mmol l^−1^ and others do not even recommend a specific target, such as the American and Canadian‘ Diabetes Associations.^64–66^

### Strengths and limitations of the study

Our systematic review on CGM in the peri-operative period was comprehensive. This study followed both a prepublished protocol and the Cochrane methodology. However, there are several limitations. First, there is a lack of RCTs and controlled studies in general. This did not allow any head-to-head comparison between CGM and alternative strategies, such as POC testing. The included studies suffered limitations specific to the observational design, such as a greater risk of bias and the presence of unadjusted confounders. Observational studies remain very common because of several advantages such as feasibility, fewer ethical concerns and larger study cohorts.^67^ Heterogeneity was observed across studies in terms of type of surgery, patients and CGM devices used. This limited the feasibility of quantitative analysis, together with the underreporting of certain outcomes. Second, the absence of individual patient data prevents an unbiased quantitative analysis. A few studies reported the use of obsolete older sensors and displayed reduced accuracy compared with more recent devices. Subgroup data based on the occurrence of diabetes and its type were rarely available, thereby reducing the ability to determine whether the use of CGM is more useful in diabetic or nondiabetic patients. Third, a majority of studies did not assess or report safety outcomes. These limitations lead to a very low certainty in the current findings.

## Conclusions

This study highlights the use of CGM devices across diverse surgical procedures and patient groups, with notable differences in observed glycaemic profiles. Device-related adverse events are uncommon and mild, whereas CGM dysfunction is relatively frequent in this specific group. Standardisation of the reporting of glucose ranges and the systematic assessment of adverse events and device dysfunction is recommended in future studies. Due to the limited number of controlled studies, the impact of CGM on postoperative glycaemic control and complications compared with POC testing remains unknown. This underscores the need for further research, including RCTs and case–control studies, to better understand the effectiveness and safety of CGM in peri-operative care.

## Supplementary Material

Supplemental Digital Content
