# Supplementary material for: Continuous peri-operative glucose monitoring in noncardiac surgery: A systematic review
Source: Eur J Anaesthesiol. 2024 Nov 7;42(2):162–71. doi: 10.1097/EJA.0000000000002095 (PMC11676603; doi:10.1097/EJA.0000000000002095)
Supplement: Supplemental Digital Content [file ejanet-42-162-s001.docx]

**SUPPLEMENTARY DATA FILE**

**Title**

Perioperative continuous glucose monitoring in non-cardiac surgery: a systematic review.

**Authors**

Alessandro Putzu, MD; Elliot Grange, MD; Raoul Schorer, MD; Eduardo Schiffer, MD; Karim Gariani, MD.

**Summary**

[**Supplementary Table S1 - PRISMA 2020 checklist** 2](#_Toc172577953)

[**Supplementary Methods S1 – Search Strategies** 5](#_Toc172577954)

[**Supplementary Table S2 – Outcomes definitions of included studies** 6](#_Toc172577955)

[**Supplementary Table S3 – Outcomes reported by included studies** 7](#_Toc172577956)

[**Supplementary Methods S2 - MINORS: Methodological items for non-randomized studies** 8](#_Toc172577957)

[**Supplementary Methods S3 - Financial and non-financial conflict of interest.** 9](#_Toc172577958)

[**Supplementary Table S4 – Deviations from study protocol** 10](#_Toc172577959)

[**Supplementary Table S5 - Major exclusion with reason** 11](#_Toc172577960)

[**Supplementary Table S6 - Additional study characteristics** 13](#_Toc172577961)

[**Supplementary Table S7 – Risk of bias results for nonrandomized trials: MINORS** 14](#_Toc172577962)

[**Supplementary Figure S1 –- Risk of bias results for randomized trials: ROB2** 15](#_Toc172577963)

[**Supplementary Table S8 – Details on conflicts of interests** 16](#_Toc172577964)

[**Supplementary Table S9 – Device related adverse events, device replacement, and device dysfunction.** 18](#_Toc172577965)

## **Supplementary Table S1 - PRISMA 2020 checklist**

Abstract checklist

| **Section and Topic** | **Item #** | **Checklist item** | **Reported (Yes/No)** |
| --- | --- | --- | --- |
| **TITLE** | | |  |
| Title | 1 | Identify the report as a systematic review. | Yes |
| **BACKGROUND** | | |  |
| Objectives | 2 | Provide an explicit statement of the main objective(s) or question(s) the review addresses. | Yes |
| **METHODS** | | |  |
| Eligibility criteria | 3 | Specify the inclusion and exclusion criteria for the review. | Partially (words limit) |
| Information sources | 4 | Specify the information sources (e.g. databases, registers) used to identify studies and the date when each was last searched. | Partially (words limit) |
| Risk of bias | 5 | Specify the methods used to assess risk of bias in the included studies. | No (words limit) |
| Synthesis of results | 6 | Specify the methods used to present and synthesise results. | No (words limit) |
| **RESULTS** | | |  |
| Included studies | 7 | Give the total number of included studies and participants and summarise relevant characteristics of studies. | Yes |
| Synthesis of results | 8 | Present results for main outcomes, preferably indicating the number of included studies and participants for each. If meta-analysis was done, report the summary estimate and confidence/credible interval. If comparing groups, indicate the direction of the effect (i.e. which group is favoured). | Yes |
| **DISCUSSION** | | |  |
| Limitations of evidence | 9 | Provide a brief summary of the limitations of the evidence included in the review (e.g. study risk of bias, inconsistency and imprecision). | Yes |
| Interpretation | 10 | Provide a general interpretation of the results and important implications. | Yes |
| **OTHER** | | |  |
| Funding | 11 | Specify the primary source of funding for the review. | No (words limit) |
| Registration | 12 | Provide the register name and registration number. | Yes |

*From:*  Page MJ, McKenzie JE, Bossuyt PM, Boutron I, Hoffmann TC, Mulrow CD, et al. The PRISMA 2020 statement: an updated guideline for reporting systematic reviews. BMJ 2021;372:n71. doi: 10.1136/bmj.n71. For more information, visit: http://www.prisma-statement.org/

Manuscript checklist

| **Section and Topic** | **Item #** | **Checklist item** | **Location where item is reported** |
| --- | --- | --- | --- |
| **TITLE** | | |  |
| Title | 1 | Identify the report as a systematic review. | 1 |
| **ABSTRACT** | | |  |
| Abstract | 2 | See the PRISMA 2020 for Abstracts checklist. | Supplement |
| **INTRODUCTION** | | |  |
| Rationale | 3 | Describe the rationale for the review in the context of existing knowledge. | 3-4 |
| Objectives | 4 | Provide an explicit statement of the objective(s) or question(s) the review addresses. | 4 |
| **METHODS** | | |  |
| Eligibility criteria | 5 | Specify the inclusion and exclusion criteria for the review and how studies were grouped for the syntheses. | 5 |
| Information sources | 6 | Specify all databases, registers, websites, organisations, reference lists and other sources searched or consulted to identify studies. Specify the date when each source was last searched or consulted. | 5 |
| Search strategy | 7 | Present the full search strategies for all databases, registers and websites, including any filters and limits used. | 5, supplement |
| Selection process | 8 | Specify the methods used to decide whether a study met the inclusion criteria of the review, including how many reviewers screened each record and each report retrieved, whether they worked independently, and if applicable, details of automation tools used in the process. | 5-6 |
| Data collection process | 9 | Specify the methods used to collect data from reports, including how many reviewers collected data from each report, whether they worked independently, any processes for obtaining or confirming data from study investigators, and if applicable, details of automation tools used in the process. | 6 |
| Data items | 10a | List and define all outcomes for which data were sought. Specify whether all results that were compatible with each outcome domain in each study were sought (e.g. for all measures, time points, analyses), and if not, the methods used to decide which results to collect. | 6, supplement |
|  | 10b | List and define all other variables for which data were sought (e.g. participant and intervention characteristics, funding sources). Describe any assumptions made about any missing or unclear information. | 6, supplement |
| Study risk of bias assessment | 11 | Specify the methods used to assess risk of bias in the included studies, including details of the tool(s) used, how many reviewers assessed each study and whether they worked independently, and if applicable, details of automation tools used in the process. | 7, supplement |
| Effect measures | 12 | Specify for each outcome the effect measure(s) (e.g. risk ratio, mean difference) used in the synthesis or presentation of results. | NA |
| Synthesis methods | 13a | Describe the processes used to decide which studies were eligible for each synthesis (e.g. tabulating the study intervention characteristics and comparing against the planned groups for each synthesis (item #5)). | 7 |
|  | 13b | Describe any methods required to prepare the data for presentation or synthesis, such as handling of missing summary statistics, or data conversions. | 6 |
|  | 13c | Describe any methods used to tabulate or visually display results of individual studies and syntheses. | NA |
|  | 13d | Describe any methods used to synthesize results and provide a rationale for the choice(s). If meta-analysis was performed, describe the model(s), method(s) to identify the presence and extent of statistical heterogeneity, and software package(s) used. | NA |
|  | 13e | Describe any methods used to explore possible causes of heterogeneity among study results (e.g. subgroup analysis, meta-regression). | 7, supplement |
|  | 13f | Describe any sensitivity analyses conducted to assess robustness of the synthesized results. | Supplement |
| Reporting bias assessment | 14 | Describe any methods used to assess risk of bias due to missing results in a synthesis (arising from reporting biases). | NA |
| Certainty assessment | 15 | Describe any methods used to assess certainty (or confidence) in the body of evidence for an outcome. | NA |
| **RESULTS** | | |  |
| Study selection | 16a | Describe the results of the search and selection process, from the number of records identified in the search to the number of studies included in the review, ideally using a flow diagram. | 8 |
|  | 16b | Cite studies that might appear to meet the inclusion criteria, but which were excluded, and explain why they were excluded. | 8, supplement, Figure 1 |
| Study characteristics | 17 | Cite each included study and present its characteristics. | 8 |
| Risk of bias in studies | 18 | Present assessments of risk of bias for each included study. | 9, supplement |
| Results of individual studies | 19 | For all outcomes, present, for each study: (a) summary statistics for each group (where appropriate) and (b) an effect estimate and its precision (e.g. confidence/credible interval), ideally using structured tables or plots. | 9-11, Table 2, supplement |
| Results of syntheses | 20a | For each synthesis, briefly summarise the characteristics and risk of bias among contributing studies. | 9-11, Table 2, supplement |
|  | 20b | Present results of all statistical syntheses conducted. If meta-analysis was done, present for each the summary estimate and its precision (e.g. confidence/credible interval) and measures of statistical heterogeneity. If comparing groups, describe the direction of the effect. | 9-11, Table 2, supplement |
|  | 20c | Present results of all investigations of possible causes of heterogeneity among study results. | NA |
|  | 20d | Present results of all sensitivity analyses conducted to assess the robustness of the synthesized results. | NA |
| Reporting biases | 21 | Present assessments of risk of bias due to missing results (arising from reporting biases) for each synthesis assessed. | NA |
| Certainty of evidence | 22 | Present assessments of certainty (or confidence) in the body of evidence for each outcome assessed. | NA |
| **DISCUSSION** | | |  |
| Discussion | 23a | Provide a general interpretation of the results in the context of other evidence. | 12-15 |
|  | 23b | Discuss any limitations of the evidence included in the review. | 16 |
|  | 23c | Discuss any limitations of the review processes used. | 16 |
|  | 23d | Discuss implications of the results for practice, policy, and future research. | 12-16 |
| **OTHER INFORMATION** | | |  |
| Registration and protocol | 24a | Provide registration information for the review, including register name and registration number, or state that the review was not registered. | 5 |
|  | 24b | Indicate where the review protocol can be accessed, or state that a protocol was not prepared. | 5 |
|  | 24c | Describe and explain any amendments to information provided at registration or in the protocol. | Supplement |
| Support | 25 | Describe sources of financial or non-financial support for the review, and the role of the funders or sponsors in the review. | 5 |
| Competing interests | 26 | Declare any competing interests of review authors. | 5 |
| Availability of data, code and other materials | 27 | Report which of the following are publicly available and where they can be found: template data collection forms; data extracted from included studies; data used for all analyses; analytic code; any other materials used in the review. | 18 |

*NA, not applicable*

*From:*  Page MJ, McKenzie JE, Bossuyt PM, Boutron I, Hoffmann TC, Mulrow CD, et al. The PRISMA 2020 statement: an updated guideline for reporting systematic reviews. BMJ 2021;372:n71. doi: 10.1136/bmj.n71. For more information, visit: http://www.prisma-statement.org/

## **Supplementary Methods S1 – Search Strategies**

PubMed

(“CGM”[tiab] OR (“continuous glucose”[tiab] AND (monitor*[tiab] OR “sensing”[tiab] OR sensor*[tiab])) OR “artificial pancreas” OR “closed loop insulin”[tiab:~5]) AND (surgery[tiab] OR surgical[tiab] OR surg*[tiab] OR operative[tiab] OR perioperative[tiab] OR preoperative[tiab] OR postoperative[tiab] OR intraoperative[tiab] OR anesthesia[tiab] OR anaesthesia[tiab])

EMBASE

(“CGM”:ab,ti OR (“continuous glucose”:ab,ti AND (monitor*:ab,ti OR “sensing”:ab,ti OR sensor*:ab,ti)) OR “artificial pancreas”:ab,ti OR (“closed loop”:ab,ti AND insulin:ab,ti)) AND (surgery:ab,ti OR surgical:ab,ti OR surg*:ab,ti OR operative:ab,ti OR perioperative:ab,ti OR preoperative:ab,ti OR postoperative:ab,ti OR intraoperative:ab,ti OR anesthesia:ab,ti OR anaesthesia:ab,ti)

CENTRAL

(“CGM” OR (“continuous glucose” AND (monitor* OR “sensing” OR sensor*)) OR “artificial pancreas” OR (“closed loop” AND insulin)) AND (surgery OR surgical OR surg* OR operative OR perioperative OR preoperative OR postoperative OR intraoperative OR anesthesia OR anaesthesia)

## **Supplementary Table S2 – Outcomes definitions of included studies**

| **Study** | **TIR** | **TBR** | **TAR** |
| --- | --- | --- | --- |
| Carlsson 2023 | 3.9–10.0 mmol/L (70–180 mg/dL) | < 3.9 mmol/L (70 mg/dL) | NA |
| Fagher 2023 | 4.0–10.0 mmol/l (72–180 mg/dl) | < 4 mmol/L (72 mg/dL) | > 10 mmol/l (180 mg/dL) |
| Farmanov 2024 | 4.3-11.6 mmol/L (77–209 mg/dL) | NA | NA |
| Hagerf 2023 | 3.9–10.0 mmol/L (70–180 mg/dL) | < 3.9 mmol/L (70 mg/dL) | 10-13.9 mmol/l + >13.9 mmol/l |
| Hagerf 2024 | 3.9–10.0 mmol/L (70–180 mg/dL) | NA | NA |
| Herzig 2022 | 3.9–10.0 mmol/L (70–180 mg/dL) | < 3.9 mmol/L (70 mg/dL) | > 10 mmol/l (180 mg/dL) |
| Jabor 2023 | NA | NA | > 10 mmol/l (180 mg/dL) |
| Jo 2022 | NA | < 3.9 mmol/L (70 mg/dL) | 10-13.9 mmol/l (181-250 mg/dL) |
| Kim 2022 | 3.9–10.0 mmol/L (70–180 mg/dL) | < 3.9 mmol/L (70 mg/dL) | > 10 mmol/l (180 mg/dL) |
| Krutkyte 2023* | 5.6–10.0 mmol/L (101–180 mg/dL) | < 3.9 mmol/L (70 mg/dL) | > 10 mmol/l (180 mg/dL) |
| Leung 2023 | NA | NA | > 10 mmol/l (180 mg/dL) |
| Maeda 2019 | NA | NA | NA |
| Mao 2021 | 3.9–10.0 mmol/L (70–180 mg/dL) | < 3.9 mmol/L (70 mg/dL) | > 10 mmol/l (180 mg/dL) |
| Mittal 2015 | 3.9–7,8 mmol/L (70–140 mg/dL) | < 3.9 mmol/L (70 mg/dL) | > 7,8 mmol/l (140 mg/dL) |
| Munekage 2016 | NA | NA | NA |
| Shaban 2023 | 4 and 11 mmol/L (72–198 mg/dL) | NA | NA |
| Turquetil 2021 | 3.9–7.8 mmol/L (70–140 mg/dL) | < 3.9 mmol/L (70 mg/dL) | > 7.8 mmol/L (140 mg/dL) |
| Wang 2021 | NA | NA | NA |
| Wysocki 2019 | 3.9–10.0 mmol/L (70–180 mg/dL) | < 3.9 mmol/L (70 mg/dL) | > 6.7 mmol/l (121 mg/dL) |
| Wysocki 2024 | 3.9–6.7 mmol/L (70–120 mg/dL) | < 3.9 mmol/L (70 mg/dL) | > 6.7 mmol/l (120 mg/dL) |
| Yin 2022 | 3.9–10.0 mmol/L (70–180 mg/dL) | < 3.9 mmol/L (70 mg/dL) | > 10 mmol/l (180 mg/dL) |
| Yip 2014 | 4–7 mmol/L | NA | NA |
| Yong Jin 2019 | NA | NA | NA |
| Zhang 2024 | 3.9–7.8 mmol/L (70–140 mg/dL) | < 3.9 mmol/L (70 mg/dL) | > 7.8 mmol/L (140 mg/dL) |

NA, not applicable or not reported; TIR, time in range; TBR, time below range; TAR, time above range.

*Post hoc study of ‘Herzig 2022’.

## **Supplementary Table S3 – Outcomes reported by included studies**

1. Any outcome

| **Study** | **Any glycemic outcome** | **DRAE** | **Device dysfunction** | **Comparative study** | **Any predefined clinical outcome** |
| --- | --- | --- | --- | --- | --- |
| Carlsson 2023 | Yes | Yes | Yes | No | NA |
| Fagher 2023 | Yes | No | Yes | Yes | No |
| Farmanov 2024 | Yes | No | No | Yes | No |
| Hagerf 2023 | Yes | No | No | Yes | No |
| Hagerf 2024 | Yes | Yes | Yes | Yes | No |
| Herzig 2022 | Yes | Yes | Yes | Yes | Yes |
| Jabor 2023 | Yes | No | Yes | No | NA |
| Jo 2022 | Yes | No | No | No | NA |
| Kim 2022 | Yes | Yes | Yes | No | NA |
| Leung 2023 | Yes | No | No | No | NA |
| Maeda 2019 | Yes | No | No | No | NA |
| Mao 2021 | Yes | No | No | No | NA |
| Mittal 2015 | Yes | No | Yes | No | NA |
| Munekage 2016 | Yes | No | Yes | Yes | No |
| Poljakova 2013 | No | Yes | Yes | No | NA |
| Price 2023 | No | Yes | Yes | No | NA |
| Shaban 2023 | Yes | No | No | No | NA |
| Tripyla 2020 | No | Yes | Yes | No | NA |
| Turquetil 2021 | Yes | No | Yes | No | NA |
| Wang 2021 | Yes | No | No | No | NA |
| Wysocki 2019 | Yes | No | No | No | NA |
| Wysocki 2024 | Yes | No | No | No | NA |
| Yin 2022 | Yes | No | No | No | NA |
| Yip 2014 | Yes | No | No | No | NA |
| Yong Jin 2019 | Yes | No | No | No | NA |
| Zhang 2024 | Yes | Yes | Yes | No | NA |

NA, not applicable

1. Glycamic outcomes

| **Surgery** | **Population** | **Mean glucose**  **level** | **Time Below Range** | **Time In Range** | **Time Above Range** | **Coefficient of Variation** |
| --- | --- | --- | --- | --- | --- | --- |
| Bariatric surgery | DM | Kim 2022, Turquetil 2021, Wang 2021, Wysocki 2019, Wysocki 2024, Yip 2014 | Kim 2022, Turquetil 2021, Wang 2021, Wysocki 2019, Wysocki 2024 | Kim 2022, Turquetil 2021, Wang 2021, Wysocki 2019, Wysocki 2024, Yip 2014 | Kim 2022, Turquetil 2021, Wang 2021, Wysocki 2019, Wysocki 2024 | Kim 2022 |
|  | Non/pre-DM | Wysocki 2019 | Wysocki 2019 | Wysocki 2019 | Wysocki 2019 | NR |
|  | Mixed | Wysocki 2019 | Wysocki 2019 | Wysocki 2019 | Wysocki 2019 | Wysocki 2019 |
| Major surgery | DM | Carlsson 2023 | Carlsson 2023 | Carlsson 2023 | Carlsson 2023 | Carlsson 2023 |
|  | Non/pre-DM | Carlsson 2023, Mao 2021, Zhang 2024 | Carlsson 2023, Mao 2021, Zhang 2024 | Carlsson 2023, Mao 2021, Zhang 2024 | Mao 2021, Zhang 2024 | Mao 2021, Zhang 2024 |
|  | Mixed | Hagerf 2023, Hagerf 2024, Jabor 2023, Munekage 2016 | Hagerf 2023 | Hagerf 2023, Hagerf 2024 | Hagerf 2023, Jabor 2023 | Jabor 2023 |
| Pancreatic surgery | DM | Krutkyte 2023* | Krutkyte 2023* | Krutkyte 2023* | Krutkyte 2023* | Krutkyte 2023* |
|  | Non/pre-DM | Mao 2021 | Mao 2021 | Mao 2021 | Mao 2021 | Mao 2021 |
|  | Mixed | Farmanov 2024, Jabor 2023 | Farmanov 2024 | Jabor 2023 | Jabor 2023 | Farmanov 2024, Jabor 2023 |
| Transplant surgery | DM | NR | NR | Shaban 2023 | NR | NR |
|  | Non/pre-DM | NR | NR | Shaban 2023 | NR | NR |
|  | Mixed | Jabor 2023, Jo 2022, Yong Jin 2019 | Jo 2022 | Shaban 2023 | Jabor 2023, Jo 2022 | Jabor 2023, Jo 2022 |

NR, not reported; *Post hoc study of ‘Herzig 2022’.

## **Supplementary Methods S2 - MINORS: Methodological items for non-randomized studies**

The score included 12 items, the first eight being specifically for non-comparative studies (see below). The items were scored 0 if not reported; 1 when reported but inadequate; and 2 when reported and adequate. Blinding was considered non-critical for an unbiased assessment of the CGM, given the intervention's origin and the difficulty in implementation, resulting in a score of 2 when no other concerns were present. The overall methodological quality was evaluated as good for a score of 16 for non-comparative studies and 24 for comparative studies, as fair with scores 12-15 for non-comparative studies or 18-23 for comparative studies, and poor for 0-11 for non-comparative studies or 0-17 for comparative studies.


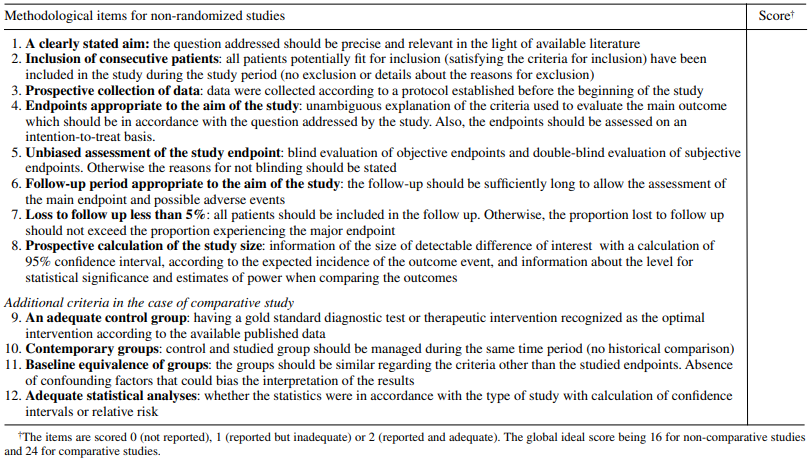


*From : Karem Slim, Emile Nini, Damien Forestier, Fabrice Kwiatkowski, Yves Panis and Jacques Chipponi, Methodological Index For Non-Randomized Studies (MINORS): Development and validation of a new instrument, ANZ J. Surg. 2003; 73: 712-716*

## **Supplementary Methods S3 - Financial and non-financial conflict of interest.**

Possible financial conflict of interest, suggested by significant commercial funding of the study or author financial (direct) conflict of interest, were assessed by two authors (AP and EG). We categorized each study as: "notable concern about conflict of interest", "no notable concern about conflict of interest", or “unclear concern about conflict of interests”. In case of unclear or not reported conflict of interest, we tried to resolve the item assessing other papers published by the study authors. We reported details on authors having conflicts of interest (e.g., lead or corresponding authors, other authors) and the stage of the trial to which they contributed (design, conduct, analysis, reporting). We reported details on funding and sponsorship of the trial and whether the role of the funding body was reported for design, conduct, analysis, and reporting.^1^

We also evaluated non-financial (indirect) conflict of interest for each author.^1, 2^ We assessed various items including multiple publications, acknowledged expert, holding a position in or consulting for a relevant committee/board/group, and obvious personal belief in relation to continuous glucose monitoring, perioperative monitoring.

*References*

1. Higgins J Thomas J, Chandler J, Cumpston M, Li T, Paige MJ, Welch VA. Cochrane Handbook for Systematic Reviews of Intervention version 6.2. *Cochrane* 2021;

2. Vandvik PO, Alhazzani W, Møller MH. Understanding conflicts of interest. *Intensive Care Medicine* Intensive Care Med; 2018; **44**: 1738–40

## **Supplementary Table S4 – Deviations from study protocol**

| **Nature of the deviation** | **Description of the deviation** | **Reason for the deviation** |
| --- | --- | --- |
| Type of data to be included | Type of data to be included | We decided to exclude preoperative data whenever possible to reduce heterogeneity, particularly due to the absence of systemic stress in response to surgery. |
| Type of data to be included | Type of data to be included in the aggregate analysis on glycaemic profile | During the review process, it was deemed appropriate to exclude from the aggregate analysis on glycemia profile studies that explicitly report non-blinding of the CGM values. This could influence perioperative glucose management compared to studies where CGM values were blinded. Furthermore, studies on pancreas transplantation were excluded due to their unique pathophysiology and perioperative glucose management. |
| Primary outcomes | Exclusion of 2 primary outcomes: the number of hypoglycaemia events and the number of hyperglycaemia events | We decided to exclude these two outcomes due to issues of underreporting and heterogeneity in the definition of outcomes, which render the pooling and external interpretation of the results unfeasible. |
| Non-primary outcomes | New tertiary outcomes: device related adverse events and device dysfunction | We have opted to incorporate these two tertiary outcomes to evaluate safety and assess any device malfunctions. |
| Risk of bias | Use of MINORS tool for non-randomized trials | We decided to use this tool since most of the studies lacked a comparative arm. |
| Subgroup analyses | Removal of subgroup analysis | Owing to the scarcity/lack of comparative studies, the heterogeneity, and the limited number of studies available, we conducted separate analyses for diabetic and non-diabetic patients, further stratifying them according to some surgical specialties. No analyses comparing these groups were performed. |
| The certainty of evidence assessment | Removal of certainty of evidence assessment | Due to the limited number/lack of comparative studies, a GRADE assessment of the evidence certainty was not conducted. Overall, the certainty of evidence was considered very low. |

## **Supplementary Table S5 - Major exclusion with reason**

| **Trial** | **Format** | **Reference** | **Reason** |
| --- | --- | --- | --- |
| Aglialoro 2011 | Abstract | Diabetes Technol Ther. 2011;13(2):200 | Mixed population (surgical and medical) |
| Amikishieva 2023 | Abstract | Diabetes Technol Ther. 2023;25(0):A234 | No outcomes of interest |
| Baba 2023 | Full-text | Sci Rep. 2023 Mar 15;13(1):4299 | Not in perioperative period |
| Bally 2018 | Abstract | Diabetes 2018 ;67(suppl 1):350 | Mixed population (surgical and medical) |
| Beauharnais 2012 | Abstract | Diabetes. 2012;61(0):A222 | Mixed population (surgical and medical) |
| Bjerkan 2024 | Full-text | Obes Res Clin Pract. 2024 Jan-Feb;18(1):9-14 | Not in perioperative period |
| Bochicchio 2017 | Full-text | J Trauma Acute Care Surg. 2017 Jun;82(6):1049-1054. | Mixed population (surgical and medical) |
| Bonner 2016 | Abstract | Am J Transplant. 2016; 16(0):422 | Not in perioperative period |
| Bublik 2023 | Abstract | Diabetes Technol Ther. 2023;25(0):A57-A58 | Not in perioperative period |
| Cummings 2023 | Full-text | Diabetes Obes Metab. 2023 Aug;25(8):2191-2202 | Not in perioperative period |
| Davis 2021 | Full-text | Diabetes Care. 2021 Jul;44(7):1641-1646 | Mixed population (surgical and medical) |
| del Rio 2014 | Full-text | J Diabetes Sci Technol. 2014 Nov;8(6):1097-104 | Not in perioperative period |
| Dorcely 2024 | Full-text | Obes Sci Pract. 2024 Jan 4;10(1):e729 | Not in perioperative period |
| Duan 2023 | Full-text | Trials. 2023 Feb 1;24(1):72. | Study protocol |
| El Bouyousfi 2019 | Abstract | Obes Surg. 2019;29(5):969 | Sample size < 10 patients |
| Elder 2017 | Full-text | Pediatr Transplant. 2017 Nov;21(7) | Pediatric study |
| Fagher 2022 | Abstract | Diabetes Technol Ther. 2022;24(0):A189 | Overlapping population |
| Farmanov 2023 | Abstract | Diabetes technology & therapeutics. , 2023, Vol.25(S2), p.A-270-A-284 | Overlapping population |
| Farmanov 2024 | Abstract | Diabetes Res Clin Pract. 2024; 209(S1) | Overlapping population |
| Ferreira 2023 | Full-text | Diabetes Technol Ther. 2023 Jul;25(7):467-475 | Not in perioperative period |
| Fujino 2013 | Full-text | Masui. 2013 Feb;62(2):140-6. | Sample size < 10 patients |
| Galindo 2020 | Full-text | Diabetes Care. 2020 Nov;43(11):2730-2735. | Mixed population (surgical and medical) |
| Hagerf 2024 | Abstract | Diabetes 2024;73(Supplement_1):1918-LB | Overlapping population |
| Hanaire 2011 | Full-text | Diabetes Technol Ther. 2011 Jun;13(6):625-30. | Not in perioperative period |
| Jin 2019 LT | Full-text | J Diabetes Res. 2019 Nov 27;2019:1757182 | Not in perioperative period |
| Joseph 2014 | Abstract | Crit Care. 2014 ;18: P439 | Mixed population (cardiac and non-cardiac surgery) |
| Kim 2023 | Full-text | Paediatr Anaesth. 2023 Oct;33(10):862-867 | Pediatric study |
| Kubota 2020 | Full-text | Gastric Cancer. 2020 Jul;23(4):699-706. doi: 10.1007/s10120-019-01036-5 | Not in perioperative period |
| Lampert 2018 | Abstract | Pancreatology. 2018;18:S1-188 | No outcomes of interest |
| Lorencio 2010 | Abstract | Intensive Care Med. 2010;36(0):S217 | No outcomes of interest |
| Lupoli 2022 | Full-text | Biomedicines. 2022 Apr 16;10(4):916 | Not in perioperative period |
| McCune 2023 | Full-text | Clin Transplant. 2023 Apr;37(4):e14920 | Not in perioperative period |
| McEachron 2021 | Full-text | Transpl Int. 2021 Jan;34(1):87-96. doi: 10.1111/tri.13762 | Not in perioperative period |
| Migdal 2020 | Full-text | J Diabetes Sci Technol. 2020 Nov;14(6):1135-1136 | Mixed population (surgical and medical) |
| Nair 2017 | Abstract | Diabetes. 2017;66(1):A229–A398 | Sample size < 10 patients |
| Nilsen 2024 | Full-text | Surg Obes Relat Dis. 2024 Jan;20(1):10-16. | Not in perioperative period |
| Pasquel 2020 | Full-text | Diabetes Care. 2020 Jun;43(6):1242-1248. | Mixed population (surgical and medical) |
| Poljakova 2014 | Abstract | Diabetes Technology & Therapeutics. 2014 Feb;A1-A162 | Overlapping population |
| Prendin 2022 | Full-text | Diabetes Obes Metab. 2022 Oct;24(10):2061-2065. | Not in perioperative period |
| Ramos-Levi 2023 | Full-text | J Clin Med. 2023 Jun 27;12(13):4295. | Not in perioperative period |
| Ri 2021 | Full-text | J Gastric Cancer. 2021 Dec;21(4):325-334 | Not in perioperative period |
| Rilo 2013 | Abstract | Transplantation. 2013 ;96(0):S8 | Not in perioperative period |
| Russell 2012 | Abstract | J Diabetes Sci Technol. 2012;6(1):A31 | Mixed population (surgical and medical) |
| Segev 2021 | Full-text | J Clin Med. 2021 Apr 27;10(9):1893 | Pediatric study |
| Segev 2021 | Full-text | J Clin Med. 2021;10:1893. | Pediatric study |
| Shaban 2021 | Abstract | [https://anzsnasm.com/12845 [accessed on 28 Sept 2023]](https://anzsnasm.com/12845%20%5baccessed%20on%2028%20Sept%202023%5d) | Overlapping population |
| Sheehan 2022 | Full-text | Diabetes Obes Metab. 2022 Jun;24(6):1021-1028. doi: 10.1111/dom.14665 | Not in perioperative period |
| Shibamoto 2022 | Full-text | Surg Today. 2022 Nov;52(11):1634-1644 | Not in perioperative period |
| Shibamoto 2023 | Full-text | Anticancer Res. 2023 Feb;43(2):857-864 | Not in perioperative period |
| Shoda 2022 | Full-text | Surg Today. 2022 Jun;52(6):889-895 | Not in perioperative period |
| Somani 2023 | Abstract | Diabetes 2023;72(S1):242 | Not in perioperative period |
| Spanakis 2022 | Full-text | Diabetes Care 2022;45:2369–2375 | Mixed population (surgical and medical) |
| Tiberi 2023 | Full-text | J Pediatr Endocrinol Metab. 2023 May 31;36(8):803-807. | Sample size < 10 patients |
| Tripyla 2020 | Abstract | Diabetes. 2020;69(0) | Overlapping population |
| Umpierrez 2022 | Abstract | Diabetologia. 2022;65:S53 | Overlapping population |
| Varshney 2019 | Abstract | HPB. 2019 ;21:S432 | Sample size < 10 patients |
| Vonderau 2021 | Full-text | Clin Transplant. 2021 Nov;35(11):e14450 | Not in perioperative period |
| Vonderau 2021 | Full-text | Clin Transplant. 2021 Nov;35(11):e14450. | Not in perioperative period |
| Wasiq 2023 | Full-text | Eur J Pediatr. 2023 Mar;182(3):1083-1087. | Pediatric study |
| Watanabe 2012 | Abstract | Clin Nutr Suppl. 2012;7(1):132-133 | Pediatric study |
| Wollersheim 2016 | Full-text | Ann Intensive Care. 2016 Dec;6(1):70 | Mixed population (surgical and medical) |
| Yamashita 2008 | Full-text | Acta Anaesthesiol Scand 2009; 53: 66–71 | Use of an artificial pancreas device |
| Yang 2019 | Abstract | HPB. 2019; 21(S2):S482 | Not in perioperative period |
| Yatabe 2011 | Full-text | Crit Care Med. 2011 Mar;39(3):575-8 | Mixed population (surgical and medical) |
| Zaimoku 2017 | Abstract | J Hepato-Biliary-Pancreatic Sci. 2017; 24(0):A261 | Sample size < 10 patients |

## **Supplementary Table S6 - Additional study characteristics**

| **Study** | **Study timeline** | **Age (years)** | **Female (%)** | **BMI** | **ASA score** | **Estimated duration of CGM monitoring (days)** | **Use of a perioperative insulin protocol** | **Blinding of CGM data** |
| --- | --- | --- | --- | --- | --- | --- | --- | --- |
| Carlsson 2023 | 2020-2021 | 68.3 ± 10.6 | 34% | 27 ± 5.4 | ASA 2: 41; ASA 3: 57; ASA 4: 2 | 4 | Yes | Yes |
| Fagher 2023 | 2017-2019 | 69.7 ± 9.8 | 47% | NR | NR | 5 | Yes | No |
| Farmanov 2024 | 2020-2022 | 65 | 62% | NR | NR | Early postoperative period (before enteral nutrition) | Yes | No |
| Hagerf 2023 | 2023 or before | 64.5 ± 18.8 | 14% | 24.35 ± 7.3 | NR | 9 | Yes | NR |
| Hagerf 2024 | 2022- | 60 ± 16.7 | 38% | 26.9 ± 4.5 | NR | 7 (length of ICU stay) | Yes | NA |
| Herzig 2022 | 2020-2021 | 68.45 ± 12.3 | 34% | 30.1 ± 5.8 | NR | 9 | No | Yes |
| Jabor 2023 | NR | NR | NR | NR | NR | 9 | NR | NR |
| Jo 2022 | 2021-2022 | NR | NR | NR | NR | 28 | NR | NR |
| Kim 2022 | 2020-2021 | 47.2 ± 9.1 | 75% | 37.2 ± 5.7 | NR | 6 | NR | NR |
| Krutkyte 2023* | 2020-2021 | 69.9 ± 17.6 | 54% | 27.9 ± 3 | NR | 12 | Yes | Yes |
| Leung 2023 | 2020-2022 | 69.1 ± 6.6 | 82% | 28.8 ± 3.8 | NR | 4 | NA | No |
| Maeda 2019 | 2016-2017 | 69.6 ± 6.7 | 75% | 26.1 ± 4.1 | NR | 6 | Yes | No |
| Mao 2021 | 2020 | 57.1 ±13.4 | 61% | 21.21±2.51 | NR | 10 | No | Yes |
| Mittal 2015 | 2013-2014 | 43.7 ± 10.1 | NR | NR | NR | 6 | Yes | Yes |
| Munekage 2016 | 2015 or before | 69 ± 9 | 27% | 24 ± 5 | NR | NR | Yes | Yes |
| Poljakova 2013 | 2010-2011 | 69 | 47% | NR | NR | 3 | NR | No |
| Price 2023 | 2022 or before | 61.8 ± 10.8 | 43% | 32.5 ± 6.9 | NR | NR | No | Yes |
| Shaban 2023 | 2020-2021 | 60 ± 11.1 | 29% | 28.5 ± 3.0 | NR | 14 | No | Yes |
| Tripyla 2020 | 2019-2020 | 69 ± 13 | 25% | 27.3 ± 6.6 | NR | > 7 | NR | Yes |
| Turquetil 2021 | 2016-2017 | 47 ± 2 | 68% | 41.9 ± 5.3 | NR | 14 | NR | Yes |
| Hagerf 2023 | 2023 or before | 64.5 ± 18.8 | 14% | 24.35 ± 7.3 | NR | 9 | Yes | NR |
| Wang 2021 | 2015-2017 | 35.0 ± 12.6 | 51% | 41.0 ± 6.8 | NR | 6 | NR | Yes |
| Wysocki 2019 | 2019 or before | 43.1± 13.4 | 63% | 43.3 ± 5.7 | ASA 2: 87%; ASA 3: 20% | 10 | Unclear | Yes |
| Wysocki 2024 | 2020-2023 | 45 ± 10 | 70% | 47.5 ± 5.9 | ASA 2: 12%; ASA 3: 88% | 14 | NR | Yes |
| Yin 2022 | 2019 | 68.29 ± 8.48 | 31% | 25.48 ± 4.24 | NR | 5 | No | Yes |
| Yip 2014 | 2010-2012 | 42.3 ± 7.7 | 76% | 43.3 ± 5.1 | NR | 6 | NR | Yes |
| Yong Jin 2019 | 2017-2018 | 50.2 ± 9.0 | 39% | 24.0 ± 3.1 | NR | 7 | NR | NR |
| Zhang 2024 | 2022-2023 | 67.2 ± 8.9 | 32% | 22.8 ± 2.3 | NR | 10 | NR | NR |

ASA, American Society of Anesthesiologists; BMI, body mass index; CGM, continuous glucose monitoring; ICU, intensive care unit; NA, not applicable; NR, not reported. *Post hoc study of ‘Herzig 2022’.

## **Supplementary Table S7 – Risk of bias results for nonrandomized trials: MINORS**

| **Study** | **A clearly stated**  **aim** | **Inclusion of consecutive patients** | **Prospective collection of data** | **Endpoints appropriate to the aim of the study** | **Unbiased assessment of the study endpoint** | **Follow-up period appropriate to the aim of the study** | **Loss to follow-up less than 5%** | **Prospective calculation of the study size** | **An adequate control group** | **Contemporary groups** | **Baseline equivalence of groups** | **Adequate statistical analyses** | **TOTAL for non-randomized studies and comparative studies** | **Overall methodological quality** |
| --- | --- | --- | --- | --- | --- | --- | --- | --- | --- | --- | --- | --- | --- | --- |
| Carlsson 2023 | 2 | 2 | 2 | 2 | 2 | 2 | 1 | 1 |  |  |  |  | 14 | Fair |
| Fagher 2023 | 2 | 1 | 1 | 2 | 2 | 2 | 1 | 0 | 1 | 0 | 1 | 1 | 14 | Poor |
| Farmanov 2024 | 1 | 1 | 0 | 2 | 2 | 2 | 1 | 0 | 1 | 2 | 1 | 1 | 14 | Poor |
| Hagerf 2023 | 1 | 0 | 2 | 2 | 1 | 2 | 1 | 0 | 1 | 2 | 1 | 1 | 14 | Poor |
| Jabor 2023 | 2 | 0 | 0 | 2 | 2 | 2 | 0 | 0 |  |  |  |  | 8 | Poor |
| Jo 2022 | 2 | 0 | 2 | 0 | 2 | 2 | 0 | 0 |  |  |  |  | 8 | Poor |
| Kim 2022 | 2 | 1 | 2 | 2 | 2 | 2 | 2 | 2 |  |  |  |  | 15 | Fair |
| Leung 2023 | 2 | 0 | 2 | 2 | 2 | 2 | 0 | 0 |  |  |  |  | 10 | Poor |
| Maeda 2019 | 1 | 1 | 2 | 2 | 2 | 2 | 1 | 0 |  |  |  |  | 11 | Poor |
| Mao 2021 | 1 | 0 | 2 | 2 | 2 | 2 | 2 | 0 |  |  |  |  | 11 | Poor |
| Mittal 2015 | 1 | 0 | 2 | 2 | 2 | 2 | 1 | 0 |  |  |  |  | 10 | Poor |
| Munekage 2016 | 2 | 0 | 2 | 2 | 2 | 0 | 1 | 0 | 2 | 2 | 1 | 1 | 15 | Poor |
| Poljakova 2013 | 1 | 0 | 2 | 2 | 2 | 2 | 1 | 0 |  |  |  |  | 10 | Poor |
| Price 2023 | 1 | 0 | 2 | 2 | 2 | 2 | 1 | 0 |  |  |  |  | 10 | Poor |
| Shaban 2023 | 1 | 2 | 2 | 2 | 2 | 2 | 2 | 0 |  |  |  |  | 13 | Fair |
| Tripyla 2020 | 2 | 2 | 2 | 2 | 2 | 2 | 2 | 0 |  |  |  |  | 14 | Fair |
| Turquetil 2021 | 1 | 1 | 2 | 2 | 2 | 2 | 1 | 0 |  |  |  |  | 11 | Poor |
| Wang 2021 | 1 | 0 | 1 | 2 | 2 | 2 | 0 | 0 |  |  |  |  | 8 | Poor |
| Wysocki 2019 | 2 | 2 | 2 | 2 | 2 | 2 | 2 | 0 |  |  |  |  | 14 | Fair |
| Wysocki 2024 | 2 | 0 | 2 | 2 | 1 | 2 | 0 | 0 |  |  |  |  | 9 | Poor |
| Yin 2022 | 1 | 0 | 2 | 2 | 2 | 2 | 1 | 0 |  |  |  |  | 10 | Poor |
| Yip 2014 | 1 | 0 | 0 | 2 | 2 | 2 | 0 | 0 |  |  |  |  | 7 | Poor |
| Yong Jin 2019 | 1 | 1 | 0 | 2 | 2 | 2 | 0 | 0 |  |  |  |  | 8 | Poor |
| Zhang 2024 | 1 | 1 | 2 | 2 | 2 | 2 | 1 | 0 |  |  |  |  | 11 | Poor |

## **Supplementary Figure S1 –- Risk of bias results for randomized trials: ROB2**


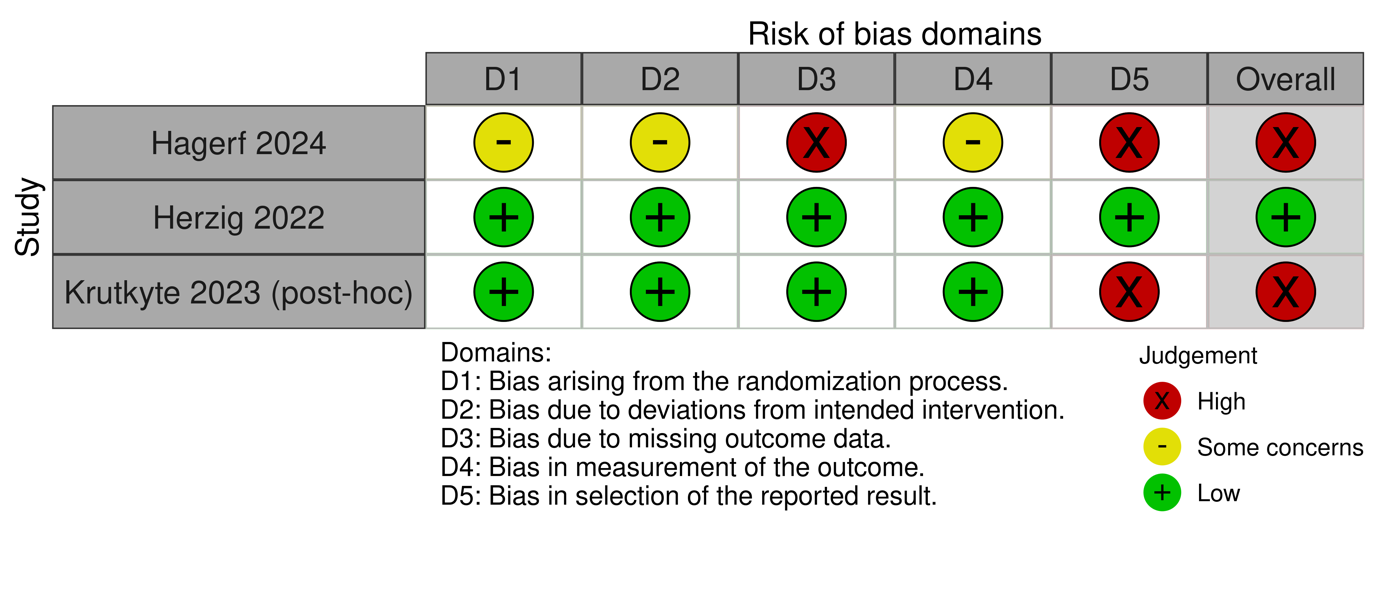


Image created using *robvis*.

Reference: McGuinness, LA, Higgins, JPT. Risk-of-bias VISualization (robvis): An R package and Shiny web app for visualizing risk-of-bias assessments. Res Syn Meth. 2020; 1- 7. https://doi.org/10.1002/jrsm.1411

## **Supplementary Table S8 – Details on conflicts of interests**

| **Trial** | **OVERALL CoI** | **Industrial funding?** | **Details on funding** | **Authors with a conflict of interest?** | **Details on authors Conflicts of interests** | **Stage of trial contribution (design, conduct, analysis, reporting)** | **Authors with a non-financial conflict of interest** |
| --- | --- | --- | --- | --- | --- | --- | --- |
| Carlsson 2023 | Notable concern about conflict of interest | No | NA | Yes | 2nd co-last author, reports direct and indirect research funding from Ferring Pharmaceuticals, Merck, Sharp & Dohme Corp. and Boehringer ingelheim outside the submitted work as well as lecture fees from Radiometer. 3rd co-last author: reports institutional research funding from Norpharma A/S outside the submitted work as well as lecture fees from Radiometer. Second author: has served as a consultant for the following CGM producing companies Medtronic, Dexcom and Abbott, and her institution has received research funding from Medtronic and Dexcom. 1st co-last author, 2nd co-last author, and 3rd co-last author: have founded a start-up company, WARD247 ApS, to pursue the regulatory and commercial activities of the WARD-project. WARD247 ApS has finalized terms for license agreement for any WARD-project software and patents. | 2^nd^ co-last author,  3^rd^ co-last author, 2^nd^ author: conception, design, conduct, reporting  Last author: guarantor of the study, conception, design, data acquisition, data analysis, interpretation and manuscript revision | Yes (2nd and last author: multiple publications and acknowledged experts) |
| Fagher 2023 | No notable concern about conflict of interest | No | NA | No | NA | NA | No |
| Farmanov 2024 | No notable concern about conflict of interest | No | NA | No | NA | NA | No |
| Hagerf 2023 | No notable concern about conflict of interest | No | NA | No | NA | NA | No |
| Hagerf 2024 | Notable concern about conflict of interest | No | NA | Yes | 13th author: a consultant to Better Therapeutics, Eoflow, GlucoTrack, Life- care, Nevro, Novo Nordisk, Sanofi, and Third- wayv | 13th author: involved in the data analysis and interpretation, discussion, and critical revision and editing of the manuscript. Provided (with another author) newly developed software support for data analysis, and reviewed and edited the manuscript. Reviewed the manuscript and approved the final version | No |
| Herzig 2022 | Notable concern about conflict of interest | Yes | Dexcom provided product support (CGM equipment). | Yes | 11th author reports receiving license fees from B. Braun and patents related to the closed-loop field and being a consultant at CamDiab Ltd. 16th author reports receiving speaker honoraria from Eli Lilly, Dexcom, and Novo Nordisk; license fees from B. Braun and Medtronic; consulting fees from Abbott Diabetes Care, patents related to the closed-loop field with the University of Cambridge and the University of Cambridge and Abbott Diabetes Care, being director and stockholder at CamDiab Ltd., and having a leadership/fiduciary role for Advanced Technologies & Treatments for Diabetes. 17th author reports advisory board fees from MSD. | 11^th^ and 16^th^ author: data analysis  17^th^ author: screening and enrolment of patients, consent, patient care, data analysis, design | Yes (1st, 11th, and last author: multiple publications and/or acknowledged experts) |
| Jabor 2023 | Notable concern about conflict of interest | No | NA | Yes | M. Haluzik is on the advisory panel of : Advisory Panel; Novo Nordisk,  Lilly Diabetes, Boehringer-Ingelheim, Research Support; Sanofi, Speaker's Bureau; Abbott, AstraZeneca. P | NR | No |
| Jo 2022 | Unclear concern about conflict of interest | Not Reported | NR | NR | NR | NR | No |
| Kim 2022 | No notable concern about conflict of interest | No | NA | No | NA | NA | No |
| Leung 2023 | No notable concern about conflict of interest | No | NA | No | NA | NA | No |
| Maeda 2019 | No notable concern about conflict of interest | No | NA | No | NA | NA | No |
| Mao 2021 | No notable concern about conflict of interest | No | NA | No | NA | NA | No |
| Mittal 2015 | No notable concern about conflict of interest | No | NA | No | NA | NA | Yes (1st and last author: multiple publications and acknowledged experts) |
| Munekage 2016 | Unclear concern about conflict of interest | Not Reported | NR | No | NA | NA | No |
| Poljakova 2013 | No notable concern about conflict of interest | No | NA | No | NA | NA | No |
| Price 2023 | Notable concern about conflict of interest | Yes | CGM devices were supplied at no cost by Dexcom and Abbott, | Yes | 3rd author discloses that he receives research support from Abbott Diabetes. | Not reported | No |
| Shaban 2023 | No notable concern about conflict of interest | No | NA | No | NA | NA | No |
| Tripyla 2020 | Notable concern about conflict of interest | Yes | The study was supported by the Dexcom External Research Program. | No | NA | NA | No |
| Turquetil 2021 | Notable concern about conflict of interest | No | NA | Yes | 3rd author has performed clinical trials as a co-investigator for Medtronic, Eli Lilly, Novo Nordisk, Sanofi and Abbott Laboratories; provided advisory services to Medtronic, Abbott, Novo Nordisk, Eli Lilly, Sanofi, Bayer, AstraZeneca, MSD, Boehringer Ingelheim and Air Liquide Sante´ International; and attended conferences organized by Novo Nordisk, Eli Lilly, Sanofi, Medtronic, Abbott, LifeScan, MSD and AstraZeneca as a contributor. Last author has done clinical trials as a co-investigator for Medtronic, Eli Lilly and Novo Nordisk; provided advisory services to Medtronic, Abbott Laboratories, Novo Nordisk, Eli Lilly and Air Liquide Sante´; attended conferences organized by Novo Nordisk, Eli Lilly, Sanofi and Medtronic as a contributor; and received investigator’s fees from Medtronic. | 3^rd^ author : data analysis, review, data collection  Last author : conception, writing, data analysis data collection, guarantor of this work (full access to the data) | Yes (2^nd^ and last author: multiple publications and/or acknowledged experts) |
| Wang 2021 | No notable concern about conflict of interest | No | NA | No | NA | NA | No |
| Wysocki 2019 | No notable concern about conflict of interest | No | NA | No | NA | NA | Yes (1st and last author: multiple publications and acknowledged experts) |
| Wysocki 2024 | No notable concern about conflict of interest | No notable concern about conflict of interest | No | NA | No | NA | Yes (1st and last author: multiple publications and acknowledged experts) |
| Yin 2022 | No notable concern about conflict of interest | No | NA | No | NA | NA | No |
| Yip 2014 | Unclear concern about conflict of interest | No | NA | No | NA | NA | Yes (last author) |
| Yong Jin 2019 | No notable concern about conflict of interest | No | NA | No | NA | NA | No |
| Zhang 2024 | No notable concern about conflict of interest | No | NA | No | NA | NA | No |

CGM, continuous glucose monitoring; NA, not applicable; NR, not reported.

## **Supplementary Table S9 – Device related adverse events, device replacement, and device dysfunction.**

|  | DRAEs | Localization (number of patients) | Replacement (number of events) | Device dysfunction |
| --- | --- | --- | --- | --- |

| **Study** | Yes | Patients | % | Arm | Abdomen | Infraclavicular | Yes | Patients | Yes | Patients | % |
| --- | --- | --- | --- | --- | --- | --- | --- | --- | --- | --- | --- |
| Carlsson 2023 | 0 | 70 | 0,00% | 79 | 0 | 0 | NR | NR | 9 | 85 | 10.59% |
| Fagher 2023 | NR | NR | NR | 100 | 0 | 0 | NR | NR | 12 | 100 | 12.00% |
| Farmanov 2024 | NR | NR | NR | NR | NR | NR | NR | NR | NR | NR | NR |
| Hagerf 2023 | NR | NR | NR | 7 | 0 | 7 | NR | NR | NR | NR | NR |
| Hagerf 2024 | 2 | 65 | 3.08% | 0 | 0 | 65 | NR | NR | 2 | 65 | 3.08% |
| Herzig 2022 | 0 | 44 | 0.00% | NR | NR | NR | 3 | 23 | 2 | 22 | 9.09% |
| Jabor 2023 | NR | NR | NR | 0 | 0 | 61 | 3 | 61 | 3 | 61 | 4.92% |
| Jo 2022 | NR | NR | NR | NR | NR | NR | NR | NR | NR | NR | NR |
| Kim 2022 | 0 | 20 | 0,00% | NR | NR | NR | 0 | 20 | 0 | 20 | 0.00% |
| Leung 2023 | NR | NR | NR | 83 | 0 | 0 | NR | NR | NR | NR | NR |
| Maeda 2019 | NR | NR | NR | 0 | 26 | 0 | NR | NR | NR | NR | NR |
| Mao 2021 | NR | NR | NR | 18 | 0 | 0 | NR | NR | NR | NR | NR |
| Mittal 2015 | NR | NR | NR | 0 | 30 | 0 | 0 | 30 | 4 | 30 | 13.33% |
| Munekage 2016 | NR | NR | NR | 19 | 0 | 0 | 0 | 19 | 1 | 19 | 5.26% |
| Poljakova 2013 | 0 | 17 | 0,00% | NR | NR | NR | 0 | 17 | 0 | 17 | 0.00% |
| Price 2023 | 0 | 76 | 0,00% | 76 | 0 | 0 | NR | NR | 12 | 94 | 12.77% |
| Shaban 2023 | NR | NR | NR | 21 | 0 | 0 | NR | NR | NR | NR | NR |
| Tripyla 2020 | 2 | 20 | 10,00% | 20 | 0 | 0 | 7 | 20 | 7 | 20 | 35% |
| Turquetil 2021 | NR | NR | NR | NR | NR | NR | NR | NR | 2 | 33 | 6.06% |
| Wang 2021 | NR | NR | NR | NR | NR | NR | NR | NR | NR | NR | NR |
| Wysocki 2019 | NR | NR | NR | 32 | 0 | 0 | NR | NR | NR | NR | NR |
| Wysocki 2024 | NR | NR | NR | 32 | 0 | 0 | NR | NR | NR | NR | NR |
| Yin 2022 | NR | NR | NR | NR | NR | NR | NR | NR | NR | NR | NR |
| Yip 2014 | NR | NR | NR | 0 | 31 | 0 | NR | NR | NR | NR | NR |
| Yong Jin 2019 | NR | NR | NR | 31 | 0 | 0 | NR | NR | NR | NR | NR |
| Zhang 2024 | 2 | 42 | 4.76% | NR | NR | NR | NR | NR | 2 | 42 | 4.76% |
| **TOTAL:** | **6** | **354** | **1.69%** | **486** | **87** | **133** | **12** | **199** | **56** | **608** | **9.21%** |

DRAE, device-related adverse event; NR, not reported.
